# Alarm of non-communicable disease in Iran: Kavar cohort profile, baseline and 18-month follow up results from a prospective population-based study in urban area

**DOI:** 10.1371/journal.pone.0260227

**Published:** 2022-01-27

**Authors:** Ali Reza Safarpour, Mohammad Reza Fattahi, Ramin Niknam, Firoozeh Tarkesh, Vahid Mohammadkarimi, Shahrokh Sadeghi Boogar, Elham Abbasi, Firoozeh Abtahi, Gholam Reza Sivandzadeh, Fardad Ejtehadi, Mohammad Afshar, Seyed Ali Shamsnia, Nasim Niknejad

**Affiliations:** 1 Gastroenterohepatology Research Center, Shiraz University of Medical Sciences, Shiraz, Iran; 2 Department of Internal Medicine, School of Medicine, Shiraz University of Medical Sciences, Shiraz, Iran; 3 Cardiovascular Research Center, Shiraz University of Medical Sciences, Shiraz, Iran; 4 Kavar Health System Network, Kavar County, Shiraz University of Medical Sciences, Shiraz, Iran; 5 School of Population and Public Health, The University of British Columbia, Vancouver, Canada; Howard University, UNITED STATES

## Abstract

The PERSIAN Kavar cohort study (PKCS) aims to investigate the prevalence, trends, and relevant prognostic risk factors of non-communicable diseases in participants aged 35–70 years living in the urban area of Kavar County. Kavar County is located at the center of Fars province in the southwest of Iran. Overall, 5236 adults aged 35–70 years old were invited to participate in the PKCS. From whom, 4997 people comprising 2419 men and 2578 women met the inclusion criteria and were recruited in the study (participation rate: 95.4%). This study is aimed to follow participants for at least 10 years; it is designed to perform all procedures similar to the primary phase including biological sampling, laboratory tests, physical examinations, and collecting general, nutritional, and medical data at the 5^th^ and 10^th^ years of follow-up. In addition, participants are annually followed-up by phone to acquire data on the history of hospitalization, any major diagnosis or death. At the enrollment phase, trained interviewers were responsible for obtaining general, nutritional, and medical data utilizing a 482-item questionnaire. The results of the baseline phase of this study show that the overweight category was the most prevalent BMI category among the registered participants (n = 2005, 40.14%). Also, almost one-third of Kavar adult population suffered from metabolic syndrome at the baseline phase (n = 1664, 33.30%). The rate of eighteen-month follow-up response was 100% in the PKCS. Hypertension (n = 116, 2.32%), cardiovascular outcomes (n = 33, 0.66%), and diabetes (n = 32, 0.64%) were the most prevalent new-onset NCDs during eighteen months of follow-up in the participants.

## Introduction

A few decades ago, communicable diseases were the leading cause of death in developing countries [1]. The emergence of vaccines and the improvements in health care facilities were two effective strategies which brought about a revolution in population health and decreased communicable diseases mortality. Subsequently, the mortality associated with communicable, maternal, neonatal, and nutritional disorders reduced from the annual rate of 16.1 million deaths in 1990 to 11.8 million deaths in 2013 [2, 3].

From that time onwards, non-communicable diseases (NCDs) have become the most prevalent public health issue worldwide; in 1990, the prevalence of NCDs worldwide was 57% which rose to 71% in 2018 [3, 4]. Transition to modern lifestyle and higher life expectancy can increase NCDs risk factors and subsequently cause an outbreak in NCDs incidence rate [5].

In Iran, total NCD deaths were 304,400 in 2016 which accounted for 82% of all deaths [6]. Developments in the health care system, namely the advancements in controlling infectious diseases, have extended life expectancy in the Iranian population [7]. On the other hand, the modernization of Iranian’s lifestyle, particularly the acquisition of western dietary patterns and low physical activity, accompanied by the extended life span put a higher percentage of Iranians at risk for NCDs [7–9].

Population-based longitudinal studies on NCDs are of prominent importance in understanding the prevalence, trends, and relevant prognostic risk factors of NCDs. The aforementioned studies can be fundamental for tailoring an evidence-based preventive tool for controlling NCDs [10].

The unique lifestyle of Iranians together with the high diversity of ethnicities necessitates performing separate longitudinal studies on different Iranian ethnic groups around the country. The sub-national population-based studies on NCDs in Iran are scarce. The Golestan Cohort Study (GCS) is the biggest prospective population-based study in the Middle East aimed to investigate the main risk factors of upper gastrointestinal tract cancers in around 50,000 participants residing northeast of Iran [11]. GCS showed that more than 80% of deaths in the studied population are due to NCDs e.g. cardiovascular disease and cancers [12].

The PERSIAN (Prospective Epidemiological Research Studies in IrAN) cohort study, which is originally designed based on GCS, is the largest population-based cohort study in Iran aimed to determine the prevalence of NCDs and their potential risk factors in 180,000 adults 35–70 years old [13]. In 2012, the PERSIAN cohort study commenced data sampling in 18 centers distributed in different regions of Iran. The Ministry of Health and Medical Education (MOHME) has provided a standard instruction to all branches of the PERSIAN cohort study to standardize and unify the sampling methods, data collection, and data entry methods in all centers [14].

According to the results of previous studies [15, 16], the high prevalence of metabolic syndrome (Mets) in Kavar adults necessitated performing a longitudinal study to investigate this population regarding NCDs. Therefore, the PERSIAN Kavar Cohort Study has been implemented to track the eligible residents of Kavar city. The study is aimed to follow participants for at least 10 years post enrollment.

**Fig 1:**
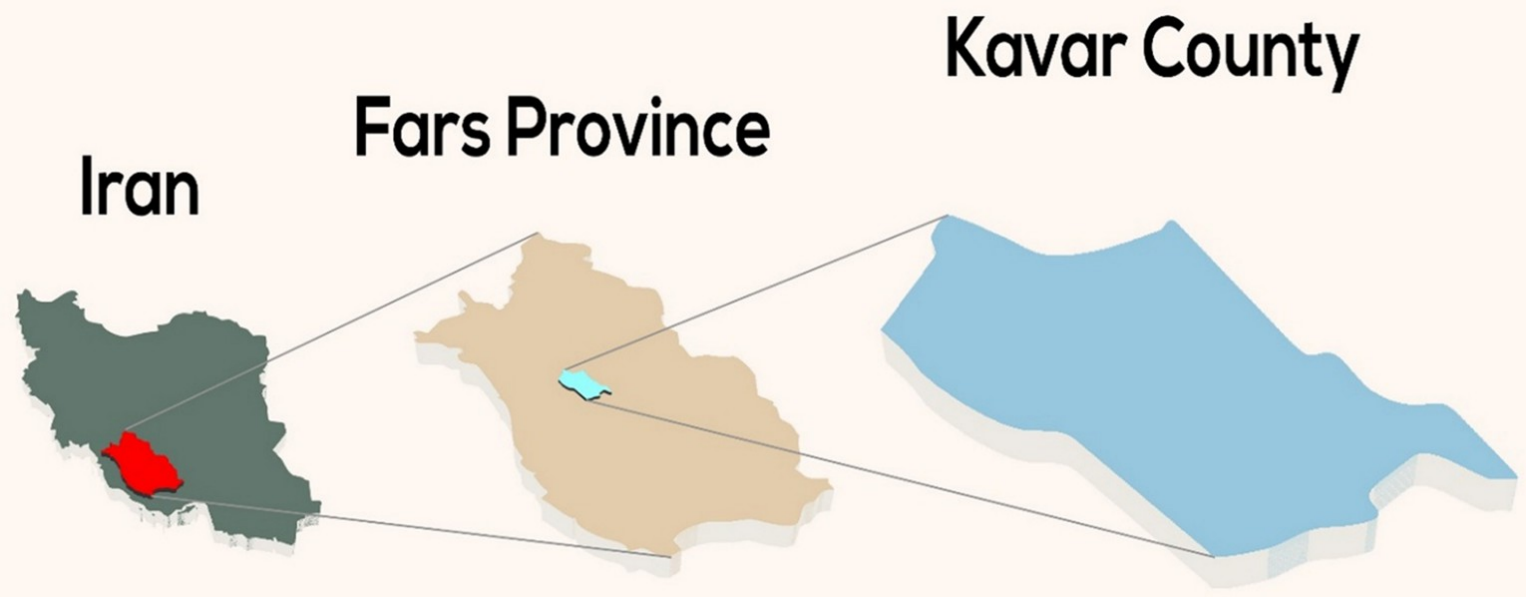
Location of Kavar County in Iran.

Kavar city, the capital of Kavar County, is located at the center of Fars province in the southwest of Iran (Fig 1). Overall, 31352 people dominantly from Fars, Turk, and Arab ethnicities reside in the urban area of Kavar county.

## Material and methods

### Cohort description

The PERSIAN Kavar Cohort Study tracks the eligible residents of Kavar city for at least 10 years post enrollment. The baseline phase of the PERSIAN Kavar cohort study (PKCS) commenced on 26/10/2017 and completed on 6/2/2019 by a team of twenty health professionals and trained staff comprised of one epidemiologist (one of the Principal Investigators), six specialist physicians including one other Principal Investigator (PI), and also three lab technicians and ten trained staff responsible for data entry including four staff for general questionnaire, two staff for medical questionnaire, and four staff for nutritional questionnaire.

From 21/05/2016 until 26/10/2017, preparations including providing the laboratory with the required equipment, and standardization of interview and central freezer rooms were made. Furthermore, among hundreds of local people, the staff were recruited and trained theoretically and practically during the mentioned period.

### Participants

The PIs introduced the PKCS, aims, benefits, and procedure to local people in 19 meetings arranged for city authorities, and residents’ gatherings such as Friday prayers in a year before beginning the study. In the recruitment phase, a list of residents eligible to participate in the study with their home address and home and cell phone numbers was prepared from the urban health centers database. The inclusion criteria were the age of 35 to 70 years old and being a permanent resident of Kavar. The exclusion criteria were unwillingness to participate in the study or having an intention to leave Kavar city.

A census method was applied for this study. So, a trained staff called all eligible residents by phone and explained the purpose, procedure, and benefits of participating in the PKCS. After confirming their willingness to participate, permanent residence, and ability to respond to the questions without help, they were assigned a date to attend the PKCS center. Overall, 5236 adults aged 35–70 years old were invited to participate in the PKCS; from whom 4997 people met the inclusion criteria and were recruited in the study (participation rate: 95.4%).

From people who were invited to participate in the PKCS, 239 individuals (4.6%) (114 women and 125 men with the mean (SD) age of 48.44 (8.38)) were unwilling to participate due to the following reasons: 1- not having enough time to participate (n = 60, 25%); 2- unwillingness to have an assessment of health status because they believed they were healthy enough (n = 45, 19%); 3- reluctance to give a blood sample for the biobank (n = 29, 12%), 4- having a plan of emigration from Kavar city (n = 74, 31%); 5- long time business trips (n = 31, 13%).

On recruitment days, the nationality and identity of each invitee were first confirmed by checking their national identity card and birth certificate. After identity confirmation, written consent form was signed by each participant. Afterward, the executive manager enrolled the participant in the PKCS and a cohort identification card with a unique code was issued for him/her.

An online software specifically designed for the PERSIAN cohort study was applied for the data entry in the PKCS. The outliers for quantitative parameters (e.g. age, anthropometric data, blood biochemical parameters, etc.) were defined in the software by the PIs before recruitment. On the data entry process, if any entered value exceeded or was lower than the defined cutoffs, the software automatically highlighted the parameter and asked for a correction or acceptance to save the value in the online software.

### Follow up

The PKCS is aimed to follow participants for at least 10 years. It is designed to perform all procedures similar to the primary phase including biological sampling, laboratory tests, physical examinations, and collecting general, nutritional, and medical data at the 5^th^ and 10^th^ years of follow-up.

**Fig 2:**
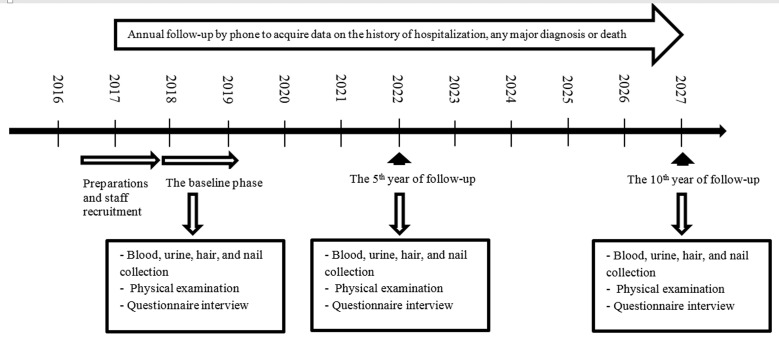
Flowchart of the PKCS.

In addition, participants are annually followed up by phone to acquire data on the history of hospitalization, any major diagnosis or death. The PKCS follow-up team makes seven-attempts to call each participant’s phone numbers on different days of the week. If a participant is inaccessible, the cohort team visit that participant in his/her address in-person. In the annual follow-up, participants are asked about any changes in their contact information or the two previously provided family members or close friends. Also, they are asked about their plans to emigrate in the coming months. Afterwards, the interviewer investigates their health status, history of hospitalization, and any newly diagnosed illnesses. Fig 2 represents the flowchart of the present cohort study.

In case a participant is dead, a team is present at his/her address to fill out a 25-item verbal autopsy form. The form includes questions about signs and symptoms, hospital documents on diagnosis and treatments, etc. Also, all the documents are scanned and uploaded to the online database. Participants who report any history of hospitalization or diagnosis of a major disease are asked to provide a copy of their medical records. Additionally, a cardiologist is responsible for confirming cardiovascular outcomes. Later the medical documents are separately assessed by two internists and each internist will assign an ICD-10 code to the documents of the dead or alive participants to indicate their diagnosis. When the ICD-10 codes are different, the final diagnosis is made by a third expert internist who is more senior. This protocol will be continued until the end of the follow-up phase.

### Ethics

The design and implementation of this study have been approved by the Ethics Committee of Shiraz University of Medical Sciences (IR.SUMS.REC.1396.S436).

### Data collection

All the data and samples collected at the baseline phase of the PKCS are shown in Table 1.

**Table 1:** Data and samples collected at the baseline phase of the PKCS.

Parameters	Classification	Measures
**Biological samples**	Blood sample	Complete blood count; fasting blood sugar; total cholesterol; high-density lipoprotein cholesterol; triglycerides; alanine transaminase; aspartate transaminase; alkaline phosphatase; γ-glutamyl transpeptidase; blood urea nitrogen; creatinine
	Urine sample	Urine PH, color, appearance, and specific gravity; presence of blood, protein, glucose, bilirubin, ketones, bacteria, epithelial, and mucus
	Hair and nail samples	These samples were placed in separate aluminum foils in codified zip-lock bags with added humidity absorber and then were stored at room temperature.
**Interview-administered questionnaires**	General	Demographic factors; socioeconomic status; occupational status and history, fuel exposures; lifestyle; sleep and circadian rhythm; physical activity; cell phone use; toxin and pesticide exposure
	Medical	Disease history, medication history, reproductive history (women), family medical history, oral and dental health, personal habits (smoking, alcohol and drug use)
	Nutritional	Food frequency questionnaire; dietary habits; food preparation and storage techniques
**physical examination**		Anthropometric measurements (Height (cm), weight (kg), waist circumference (cm), hip circumference (cm), wrist circumference (cm)); systolic and diastolic blood pressure; pulse rate measurement; oral health examination

### Bio-specimen ascertainment

This process is explained in S1 Box. Dyslipidemia was measured based on the definition of Moradinazar et al. [17].

### Physical examination

Physical examination comprised of anthropometric measurements, systolic and diastolic blood pressure and pulse rate measurement, and oral health examination was performed by a physician and trained staff. Anthropometric measurements including weight, height, and waist, hip, and wrist circumferences were done according to the US National Institutes of Health protocols [18].

Furthermore, blood pressure was measured in a sitting position after resting for five minutes. Systolic and diastolic blood pressures were measured twice in both arms with a fifteen-minute interval. Additionally, the 12-lead electrocardiograms (EKG) were carried out for all participants and then the results were interpreted by a cardiologist.

### Interview

After bio-specimen sampling and physical examination, a simple breakfast was given to participants. Then the executive manager guided participants to the next stations to prevent over-crowding. After breakfast, people were invited to the interview room for obtaining general, nutritional, and medical data through a 482-item questionnaire. Two trained staff were responsible for medical interview, four trained staff acquired nutritional data and four trained interviewers filled the general questionnaire.

#### General questions

General questions were designed to collect information about demographic variables including gender, age, ethnicity, residence, education, marital status, socio-economic status, occupation history, fuel, heating and home condition, lifestyle, sleep, physical activity, cell phone use, and contact with pesticides. Wealth score index (WSI) was estimated by performing multiple correspondence analysis (MCA) on the following variables: access to a freezer, washing machine, dishwasher, vacuum cleaner, computer, internet, motorcycle, car (no access, access to a car with a price of <50 million Tomans (national currency in Iran), and access to a car with a price of >50 million Tomans), color TV type (no color Tv or regular color Tv vs. Plasma color Tv), owning a cell phone, PC or laptop, international trips in lifetime (never, just pilgrimage, both pilgrimage or non-pilgrimage trips) [19, 20]. Then the participants were stratified into five quantiles in terms of WSI; individuals in the first quantile had the highest socio-economic status and those in the fifth quantile had the lowest socio-economic status.

#### Medical questions

Medical questions were comprised of disease history (e.g. diabetes mellitus, hypertension, heart disease, stroke, cardiovascular disease, kidney stones, gallbladder stone, asthma, tuberculosis, rheumatic disease, cancers, depression, psychological disorders, mental disease, lupus, multiple sclerosis, gestational diabetes mellitus, hypertensive disorders of pregnancy, etc.), medication history, disease history in close relatives, history of alcohol consumption, tobacco smoking, substance use, and oral health that are well described elsewhere [21]. On interview days, all participants were asked to bring their medications which were matched with their specific stated disease.

#### Nutritional questions

The Food Frequency Questionnaire (FFQ) utilized in the PKCS was a validated semi-quantitative FFQ aimed to investigate the usual dietary intake and eating habits during the previous year that its design, food items included, and validity are published previously [22]. Four trained nutritionists performed the nutritional interview. A food album and set of scales such as different sizes of spoons, cups, bowls and dished, matchbox, and a 10×10 cardboard were utilized to facilitate understanding of portion sizes.

### Quality assessment of data

Within the enrollment phase, all data items were checked for each participant by a team of quality assessors (interviewers, executive manager, and quality control assessor) based on a standard checklist, under the supervision of an epidemiologist on a daily basis. These assessments included direct supervision of PIs, recording the trained staff’s voices and desktops, and then re-educating them based on their deficiencies. Calibration of all the measurement tools and laboratory equipment and instruments, and checking all data by statistical software to detect incompleteness, missing data, and outliers were the other activities. In addition, central team of the PERSIAN cohort monitored data via a central server.

### Statistical analysis

The variables were described using number (percentage) and mean ± standard deviation (SD). The Pearson’s Chi-square test or Fischer’s exact test were used for comparison of categorical variables, and two-sided independent samples *t* test was used for comparison of quantitative variables between the groups. The normal distribution of quantitative data was checked by the Shapiro Wilks test for normality. The P-values greater than 0.05 were considered normally distributed variables. The significance level was set at the point of <0.05. IBM SPSS Statistics for Windows (Released 2012. Version 21.0. Armonk, NY: IBM Corp) and Stata software version 13 were used for analysis.

## Results

The demographic characteristics of the studied population are shown in Table 2. In the PKCS 4997 adults including 2419 (48.41%) men and 2578 (51.59%) women aged 35 to 70 years old with the mean (SD) of age 48.18 (8.92) residing in the urban area of Kavar County were investigated. A high proportion of participants were married (91.51%). The Fars and Turk nomad ethnicities were the dominant ethnicities in Kavar urban district (96.28%). A very low percentage of adult population had academic education (8.80%). There were significant differences between men and women in age, marital status, education, and WSI (P-value<0.001).

**Table 2:** Demographic characteristics of participants in PKCS.

Variables	Men n (%)	Women n (%)	Total n (%)	P- value*
**Total Number**	2419 (48.41%)	2578 (51.59%)	4997 (100%)	
**Age range**				
35–40	557 (11.15%)	727 (14.55%)	1284 (25.70%)	<0.001
41–45	445 (8.91%)	499 (9.99%)	944 (18.89%)	
46–50	434 (8.69%)	457 (9.15%)	891 (17.83%)	
51–55	359 (7.18%)	357 (7.14%)	716 (14.33%)	
56–60	308 (6.16%)	268 (5.36%)	576 (11.53%)	
61–65	224 (4.48%)	179 (3.58%)	403 (8.06%)	
66–70	92 (1.84%)	91 (1.82%)	183 (3.66%)	
**Marital status**				
Married	2354(47.11%)	2219 (44.41%)	4573 (91.51%)	<0.001
Divorced/widowed	19 (0.38%)	280 (5.60%)	299 (5.98%)	
Single	46 (0.92%)	79 (1.58%)	125 (2.50%)	
**Ethnicity**				
Fars	1858 (37.18%)	1990 (39.82%)	3848 (77.01%)	0.54
Turk Nomad	478 (9.57%)	485 (9.71%)	963 (19.27%)	
Lor	6 (0.12%)	5 (0.10%)	11 (0.22%)	
Arab Nomad	6 (0.12%)	2 (0.04%)	8 (0.16%)	
Guilak	2 (0.04%)	3 (0.06%)	5 (0.10%)	
Other**	69 (1.38%)	93 (1.826%)	162 (3.24%)	
**Education**				
Illiterate	470 (19.4%)	1089 (42.2%)	1559 (31.20%)	<0.001
Elementary	1221 (50.5%)	1153 (44.7%)	2374(47.50%)	
High school	402 (16.6%)	222 (8.65%)	624(12.50%)	
University	326 (13.5%)	114 (4.4%)	440 (8.80%)	
**Wealth Score Index (WSI)**				
1st quintile (lowest)	371 (7.42%)	618 (12.38%)	989 (19.79%)	<0.001
2nd quintile	455 (9.10%)	561 (11.23%)	1016 (20.33%)	
3rd quintile	532 (10.65%)	469 (9.39%)	1001 (20.03%)	
4th quintile	510 (10.21%)	525 (10.51%)	1035 (20.71%)	
5th quintile (highest)	551 (11.02%)	405 (8.10%)	956 (19.13%)	

*The results of chi squared test or Fisher exacted test comparing men and women.

**Other includes Arab, Turk, Azari, Kurd and other minority ethnicities.

The anthropometric and lifestyle variables are demonstrated in Table 3. The mean (SD) of BMI was 27.46 (4.91) and the overweight category was the most prevalent BMI category (40.14%). Also, 87.53% of adults had central obesity. Most of the participants consumed a medium amount of salt daily (69.36%) and did not reuse frying oil (86.07%). Furthermore, 12.53%, 37.05%, and 23.19% of participants drank alcohol, smoked water pipe or has ever smoked cigarette, respectively. The prevalence of dyslipidemia in Kavar adults was high (60.99%) comprised of 33.73% in men and 27.26% in women. The prevalence of overweight, obesity, and elevated waist-to-hip ratios (WHR) were significantly greater in women compared to men (P-value<0.001). Significant differences were also detected between men and women in salt consumption, alcohol drinking, water pipe smoking, smoking status, dyslipidemia, and physical activity (P-value<0.001).

**Table 3:** The frequency of anthropometric indices and lifestyle indicators.

Parameters	Men n (%)	Women n (%)		Total n (%)	*P- Value
**BMI**					
Underweight	79 (1.58%)	27 (0.54%)		106 (2.12%)	<0.001
Normal	1002 (20.06%)	483 (9.67%)		1485 (29.73%)	
Overweight	979 (19.60%)	1026 (20.54%)		2005 (40.14%)	
Class I obesity	300 (6.01%)	751 (15.04%)		1051 (21.04%)	
Class II & III obesity	58 (1.16%)	290 (5.81%)		348 (6.97%)	
Waist to hip ratio (WHR) **					
Normal	579 (11.59%)	44 (0.88%)		623 (12.47%)	<0.001
Abnormal	1839 (36.82%)	2533 (50.71%)		4372 (87.53%)	
**Food consumption**					
Low salt	572 (11.45%)	678 (13.57%)		1250 (25.02%)	<0.001
Medium salt	1673 (33.48%)	1793 (35.88%)		3466 (69.36%)	
Salty	174 (3.48%)	107 (2.14%)		281 (5.62%)	
**Reused frying oil**					
No	2092 (41.87%)	2209 (44.21%)		4301 (86.07%)	0.42
Yes	327 (6.54%)	369 (7.38%)		696 (13.93%)	
**Alcohol drinking**					
No	1817 (36.37%)		2553 (51.10%)	4370 (87.47%)	<0.001
Yes	602 (12.05%)		24 (0.48%)	626 (12.53%)	
**Water pipe smoking**					
No	1466 (29.34%)		1679 (33.61%)	3145 (62.95%)	<0.001
Yes	953 (19.08%)		898 (17.97%)	1851 (37.05%)	
**Smoking status**					
No	1293 (25.89%)		2544 (50.93%)	3837 (76.82%)	<0.001
Current	762 (15.26%)		13 (0.26%)	775 (15.52%)	
Former	363 (7.27%)		20 (0.40%)	383 (7.67%)	
**Dyslipidemia**					
No	733(14.69%)		1214(24.33%)	1946(39.01%)	<0.001
Yes	1683(33.73%)		1360(27.26%)	3043(60.99%)	
Physical activity (weekly METs ***, hour per week)					
24–36.5	647 (12.95%)		374 (7.48%)	1021 (20.43%)	<0.001
36.6–44.9	1078 (21.57%)		1699 (34.00%)	2777 (55.57%)	
≥45	694 (13.89%)		505 (10.11%)	1199 (23.99%)	

* The results of chi squared test or Fisher exacted test comparing men and women.

**Waist to hip ratios of less than 0.85 and 0.90 are considered as normal amounts in women and men, respectively.

***MET: metabolic equivalent of task [25].

The prevalence of Mets (according to ATP III criteria [23]) is presented in Table 4 and in S1 Fig. Almost one-third of Kavar adult population (33.30%) suffered from Mets at the baseline phase.

**Table 4:** The prevalence of metabolic syndrome (according to ATP III criteria).

No. of Mets* criteria Participants n) %)	0	1	2	3	4	5	Having Mets (≥3 criteria)
Men	477 (19.76%)	734 (30.41%)	674 (27.92%)	394 (16.32%)	116 (4.81%)	19 (0.79%)	529 (21.92)
Women	86 (3.34%)	447 (17.37%)	905 (35.17%)	700 (27.21%)	380 (14.77%)	55 (2.14%)	1135 (44.12)
Total	563 (11.27%)	1181 (23.63%)	1579 (31.60%)	1094 (21.89%)	496 (9.93%)	74 (1.48%)	1664 (33.30)

*Metabolic syndrome.

- It should be noted that 10 participants did not give blood samples in this study. Therefore, 4987 individuals were analyzed for having metabolic syndrome.

The mean (SD) values of biochemical parameters at the primary phase are presented in S1 Table. The levels of fasting blood sugar (102.81 (33.40) *vs*. 100.95 (30.78), P-value = 0.041), total cholesterol (178.53 (36.12) *vs*. 171.49 (37.95), P-value<0.001), and high density lipoprotein (HDL) (44.90 (9.61) *vs*. 39.12 (8.37), P-value<0.001) levels were higher in women compared to men. Although, the values for hemoglobin (15.51 (1.42) *vs*. 13.57 (1.37), P-value<0.001), blood urea nitrogen (30.87 (7.87) *vs*. 26.96 (7.74), P-value<0.001), creatinine (1.19 (0.18) *vs*. 0.95 (0.15), P-value<0.001), triglyceride (159.74 (103.61) *vs*. 141.42 (79.55), P-value<0.001), serum glutamic-oxaloacetic transaminase (19.78 (11.31) *vs*. 16.73 (6.84), P-value<0.001), serum glutamate pyruvate transaminase (24.09 (15.88) *vs*. 17.03 (9.84), P-value<0.001), alkaline phosphatase (202.23 (55.71) *vs*. 195.13 (60.55), P-value<0.001), and gamma glutamyl transpeptidase (27.53 (20.91) *vs*. 21.40 (17.34), P-value<0.001) were relatively higher in men than women.

The self-report prevalence of NCDs at the primary phase is shown in S2 Table. Kidney stone (n = 1430, 28.62%), hypertension (n = 931, 18.63%), diabetes (n = 783, 15.67%), fatty liver (n = 751, 15.03%) and thyroid disorders (n = 616, 12.33%) were respectively the most common chronic diseases among the urban Kavar adult population. Hypertension (n = 607 (12.15%) *vs*. n = 324 (6.49%), P-value<0.001), diabetes (n = 498 (9.97%) *vs*. n = 285 (5.70%), P-value<0.001), fatty liver (n = 504 (10.09%) *vs*. n = 247 (4.94%), P-value<0.001), thyroid disorders (n = 510 (10.21%) *vs*. n = 106 (2.12%), P-value<0.001), rheumatic diseases (n = 188 (3.76%) *vs*. n = 65 (1.30%), P-value<0.001), gallstone (n = 181 (3.62%) *vs*. n = 53 (1.06%), P-value<0.001), chronic lung disease (n = 97 (1.94%) *vs*. n = 63 (1.26%), P-value = 0.020), cancer (total) (n = 30 (0.6%) *vs*. n = 9 (0.18%), P-value = 0.001), breast cancer (n = 16 (0.32%) *vs*. n = 0, P-value<0.001), and CNS cancer (n = 6 (0.12%) *vs*. n = 0, P-value = 0.032) had a higher self-reported prevalence in women than men. However, the prevalence of kidney stones (n = 748 (14.97%) *vs*. n = 682 (13.65%), P-value<0.001), ischemic heart disease (IHD) (n = 195 (3.90%) *vs*. n = 149 (2.98%), P-value = 0.001), and myocardial infarction (MI) (n = 40 (0.8%) *vs*. n = 17 (0.34%), P-value<0.001) were significantly higher in men than women.

The association of demographic and lifestyle variables with NCDs at the start of the study in men is reported in the S3 Table. The frequency of diabetes, hypertension, and IHD significantly varied across groups of age and BMI (P-value<0.001). Education levels also associated with the prevalence of diabetes (P-value<0.001), hypertension (P-value = 0.004), and IHD (P-value = 0.007). Moreover, the association was observed between hypercholesterolemia and IHD (P-value<0.001) and also among marital status and hypertension (P-value<0.001) and IHD (P-value = 0.03) prevalence. In addition, the prevalence of diabetes and hypertension was influenced by alcohol drinking in men (P-value<0.001).

As shown in S4 Table, the baseline prevalence of diabetes, hypertension, and IHD were significantly different in various education levels and marital status in women (P-value<0.001). Age groups also associated with the frequencies of diabetes (P-value<0.001), hypertension (P-value = 0.004), and IHD (P-value = 0.007). Moreover, BMI categories (P-value<0.001) and smoking status (P-value = 0.04) associated with diabetes and hypertension, and alcohol drinking (P-value<0.001) and hypercholesterolemia (P-value = 0.03) correlated with diabetes in this group.

The rate of eighteen-month follow-up response was 100% in the PKCS. New cases of NCDs after eighteen months of follow-up are demonstrated in S5 Table. Hypertension (n = 116, 2.32%), cardiovascular outcomes (n = 33, 0.66%) and diabetes (n = 32, 0.64%) were the most prevalent new-onset NCDs within eighteen months of follow-up in the participants. As shown in S6 Table, the prevalence of diabetes was significantly different among participants with or without hypercholesterolemia after eighteen months of follow-up (P-value = 0.03). Moreover, subjects in different groups of age (P-value<0.001), education (P-value = 0.01), marital status (P-value = 0.04), or current alcohol drinking habits (P-value = 0.04) showed distinct hypertension frequencies. Gender (P-value = 0.002), age groups (P-value = 0.002), education levels (P-value = 0.04), smoking status (P-value = 0.04), and hypercholesterolemia (P-value<0.001) significantly influenced the IHD prevalence in follow-up data.

Mortality by cause during eighteen months of follow-up is shown in S7 Table. Overall, 16 (0.32%) deaths including ten women and six men occurred within this period, and sudden cardiac death was the main cause of death in both genders. New cases of NCDs and causes of death in both S7 and S8 Tables were recorded and confirmed according to ICD-10 codes [24].

## Discussion

Currently, the Iranian population is experiencing a transition in demographic, socioeconomic, dietary, and lifestyle characteristics. This transition has resulted in an epidemic of NCDs and their related risk factors [26]. Detecting NCDs-related risk factors in the population is the key to control the ascending trend of NCDs. Prospective population-based studies such as cohort studies are the best setting for investigating NCDs’ etiologies and building a platform for experimental studies aimed to control and prevention.

Iran has an ethnic diversity that contributes to different genetic susceptibilities, lifestyle habits, and disease patterns. Therefore, performing a unified set of cohort studies at the sub-national level, such as the PERSIAN Cohort Studies, is probably the most appropriate way to reveal and compare the NCD risk factors in different ethnicities and find the best strategy for NCDs prevention and control [21]. Here, we presented the design and implementation of the PKCS, one of the PERSIAN cohort sites in the southwest of Iran (mostly Fars ethnicity), and describe baseline characteristics of the participants and the NCD’s incidence and mortality rate after 18 months of follow-up. The participation rate in this study was 95.4%, which was higher than the reported rates in other PERSIAN cohorts, such as the Ravansar Non-Communicable Disease (RaNCD) (93.2%) [27], the Guilan Cohort (PGCS) (83.2%) [28], the Hoveyzeh Cohort (HCS) (82.7%) [29], the Rafsanjan Cohort (RCS) (67.42%) [30], and the Tabari Cohort studies (TCS) (61.7%) [31].

Our primary analysis indicated that most participants were married, had less than five years of education, and suffered from overweight and central obesity. These features were similar to the subjects of the PERSIAN RaNCD [27], TCS [31], and PGCS cohort studies [28], and the Pars cohort study performing in the Valashahr district in the Fars Province [32]. In addition, the prevalence of alcohol consumption was 12.53% in the total population that is similar to the reported pooled estimates in a recent meta-analysis of Iranian population [33]. However, this was higher than the reported amount of alcohol drinking in the PERSIAN RaNCD, RCS, HCS, and TCS cohort centers [27, 29–31]. Stigma linked to the alcohol consumption and different local sensitivities among Iranians may explain these discrepancies.

Mets is a clustering of central obesity, insulin resistance, hypertension, and atherogenic dyslipidemia that correlate with atherosclerotic cardiovascular disease, diabetes mellitus, and cerebrovascular accidents [34]. In the present study, 33.3% of the total population, or 44.12% of women and 21.92% of men, had metabolic syndrome, according to the ATP III criteria. This prevalence was similar to the PERSIAN Kharameh cohort [35] and higher than the pooled estimation of around 26% reported in two separate meta-analyses of studies performed in Middle-East countries and studies comprised 472401 Iranian subjects [36, 37]. Our results were consistent with the previous meta-analysis of Iranians [36, 38] and PERSIAN RaNCD [27] and Kharameh [35] cohort studies that reported higher Mets prevalence in women. Elevated body weight and waist circumference, and low HDL cholesterol are more potential contributors to the Mets in women compared to men [39]. Moreover, multiple circumstances specific to women, such as pregnancy, oral contraceptive therapy consumption, polycystic ovary syndrome, and menopause are considered as risk factors for Mets development [40]. Therefore, education and policymaking are critical approach to improve the health status and life style of these subjects.

In the current study, kidney stones, hypertension, diabetes, fatty liver, and thyroid disorders were the most common NCDs with the prevalence of 28.62%, 18.63%, 15.67%, 15.03%, and 12.33%, respectively. Lower prevalence of these disorders was observed in the RaNCD cohort study, in which kidney stone, hypertension, fatty liver, diabetes, and thyroid disorders were detected as the most prevalent NCDs, with the prevalence of 18.20%, 16.71%, 10.26%, 10.03%, and 7.34%, respectively [27]. Moreover, the prevalence of hypertension and diabetes were 16.3% and 9.3% in the Pars cohort study in 2014 [32]. Comparing to our results, participants of the HCS study had a greater prevalence of hypertension (26.4%), diabetes (22.2%), and cardiac ischemic (13.6%) and lower prevalence of kidney stone (18.8%), fatty liver (6.7%), and thyroid disorders (5.4%) [29]. Higher frequencies of hypertension and diabetes also were reported in the PERSIAN TCS and PGCS studies [28, 31]. Similarly, a meta-analysis of published studies in 2004–2018 revealed the lowest prevalence of hypertension in Isfahan, Fars, Bushehr, Chaharmahal Bakhtiari, Hormozgan, and Kohkiloyeh and Boyerahmad provinces [41]. In another meta-analysis study, Fars had a lower prevalence of diabetes compared to Guilan, Mazandaran, and Khuzestan provinces [42]. Differences in the socioeconomic features, lifestyle and dietary patterns, access to health centers, and level of health literacy could influence the prevalence of NCDs in the different regions. In addition, in our study, all common NCDs, except kidney stones, were significantly more frequent in women than men, which was in line with the most previous PERSIAN cohort studies [27, 28, 30, 31]. However, in 2019, the global prevalence of diabetes was estimated to be 9.0% in women and 9.6% in men [43]. The age-standardized prevalence of hypertension was also slightly higher in men (31.9%) than women (30.1%) in 2010 [44].

In the present study, the crude incidence of hypertension, diabetes, and cardiovascular outcomes after eighteen months of follow-up were 2.32%, 0.64%, and 0.66%, respectively. Similarly, according to a recent study derived from the Tehran Lipid and Glucose Study, nearly 2.7% of participants develop hypertension each year [45]. In another study, the overall incidence of hypertension was estimated to be 28.7 per 1000 person-years among Asian Indians [46]. In addition, the crude incidence estimation of diagnosed diabetes among adults aged 18 years or older in the United States was 6.9 per 1000 people in 2017–2018 [47]. Therefore, designing and implementation of emergency interventions to prevent the further spread of these NCDs are necessary.

The PKCS has several strengths. The population based prospective design of PKCS can properly demonstrate the trend of NCDs and their related risk factors during long-term follow-up. Furthermore, it can be a setting for performing interventional studies for prevention and control of NCDs in vulnerable subgroups. The results of PKCS can be used as a valuable evidence for investigating the most cost-effective policies to control and prevent NCDs in the population such as designing appropriate health care for the at risk population. The biological specimen bio-bank obtained according to international standards in PKCS can provide a setting for nested-case control and genetic susceptibility studies. The PKCS is carried out on a large urban population in this area. Therefore, the results can be a good indicative of the whole population health status. Another advantage of PKCS is the registration of obtained data on an online platform, which not only increases the quality and accessibility of data, but also improves the data collection accuracy by enabling the simultaneous monitoring of principal investigators on data collection. Another unique characteristic of PKCS is the significant reduction in lost to follow-up compared to other cohorts. The concurrent registration of participants’ health records on the online nation-wide Ministry of Health comprehensive health network entitled SIB network enables PKCS to track immigrated participants and obtain their health outcomes or their detailed cause of death. Moreover, cohort studies are costly studies which are widely avoided in low resources settings such as developing countries. The PKCS can serve as a good example for effective financial and human resources management in limited resources setting which made performing cohort studies in developing countries possible. The PKCS also has some limitations. The first limitation is lack of funding for performing genetic studies to reveal NCDs related genetic susceptibilities. The second limitation is inability to take and store stool samples for future investigations on the role of gut microbiota in the pathology of NCDs. The probable risks of bias and strategies for minimizing them are mentioned in Table 5.

**Table 5:** The risks of bias in the PERSIAN Kavar Cohort Study (PKCS) and strategies for their maximum control.

	Bias risk	Strategies
**Sample size, Participation bias**	Low response rate	• Using different recruitment types:
	Lack of representativeness	• Introducing the PKCS in 19 meetings arranged for city authorities, and in residents’ gatherings such as Friday prayers
	Selection bias	• Calling the eligible residents by phone
		• Enrolling all eligible people (35–70 years of age) in the study
		• Making contact a reference person
**Dropout**	Lose of interest in or miss the PKCS	• Meticulous follow-up strategies including annual follow-up by phone to acquire data on the history of hospitalization, any major diagnosis or death. If they do not answer their phones, investigators will attend their home addresses
		• Making contact a reference person
**Response**	Data were self-reported	• The study investigators guaranteed to protect the data confidentiality
		• Careful verification of patients reports and hospital records
		• Events were checked prospectively
	Reliability of responses	• Exposure measurement before the events report (prospective approach)
		• Using reference persons to verify data on the old ages
		• Assessing consistency of information
	Missing data	• Questionnaires were completed in several attempts
		• Imputation during analyzing the data when needed

## Conclusion

According to the results of this study, there is a high prevalence of metabolic syndrome in the urban population of Iran, especially in women. If fundamental changes in lifestyle and dietary patterns will not occur, this disease can lead to dangerous and far more complicated diseases such as diabetes, high blood pressure, cirrhosis, various types of heart attacks and strokes, and cancers. It is the responsibility of the health policy makers to take immediate action to change the diet and lifestyle of the Iranian people, especially women, in order to prevent a sudden increase in the incidence rate of these diseases that can impose a huge financial burden on our society.

## Supporting information

S1 Box Biospecimen ascertainment. (DOCX)

S1 Fig Prevalence of having 0 to 5 metabolic syndrome criteria and overall metabolic syndrome according to ATP III definition. (TIF)

S1 Table Biochemical parameters at the primary phase. (DOCX)

S2 Table The self-report prevalence of NCDs at the primary phase. (DOCX)

S3 Table Association of demographic and lifestyle variables with non-communicable disease at the start of study in 2419 men. (DOCX)

S4 Table Association of demographic and lifestyle variables with non-communicable disease at the start of study in 2578 women. (DOCX)

S5 Table New cases of non-communicable diseases after 18 months of follow-up at the time of registration. (DOCX)

S6 Table Association of demographic and life style variables with outcomes. (DOCX)

S7 Table Mortality by cause during 18 months of follow-up in PKCS. (DOCX)
