# Supplementary material for: Alarm of non-communicable disease in Iran: Kavar cohort profile, baseline and 18-month follow up results from a prospective population-based study in urban area
Source: PLoS One. 2022 Jan 27;17(1):e0260227. doi: 10.1371/journal.pone.0260227 (PMC8794109; doi:10.1371/journal.pone.0260227)
Supplement: S1 Box — (DOCX) [file pone.0260227.s001.docx]

After registration, participants were guided to laboratory division at the cohort center for collecting blood, urine, hair, and nail samples. Initially, 25 ml of blood after 10-14 hours of fasting state was drawn by a professional lab technician. The blood sample was distributed to three six-milliliter EDTA tubes and one seven-milliliter clot tube. The blood biochemical analysis was performed by auto analyzer (model BT3000 Plus, Biotecnica®, Italy) using kits made by Pars Azmoon Company (Iran). The blood biochemical parameters tested in the initial laboratory analysis were as follows: fasting blood sugar (FBS), serum total cholesterol (TC), triglyceride (TG), high-density lipoprotein (HDL) cholesterol, low-density lipoprotein (LDL) cholesterol, alanine aminotransferase (ALT), asparagine aminotransferase (AST), alkaline phosphatase, gamma glutamyl transpeptidase (GGT), urea, and creatinine. The complete blood count (CBC) test was carried out by automated blood cell counter device (model XS-800i, Sysmex®, Sweden). In addition, 23 ml of blood sample was centrifuged and distributed into twelve codified 2D cryo tubes including two whole blood, two buffy coat, two serum and six plasma samples stored in -70° C freezer for further supplementary tests. Dyslipidemia was defined as having at least one abnormal lipid biomarker (i.e. triglyceride, total cholesterol, LDL cholesterol, or HDL cholesterol) or using lipid lowering medications.

Participants were also asked to give 15 ml of urine sample in a sterile collection tube. The urine samples were analyzed to reveal the color, specific gravity, appearance, PH, nitrite, bilirubin, urobilinogen, protein, glucose, and blood content. Also, 2 ml of urine samples were stored for future supplementary tests.

For hair and nail sampling, people were asked not to color their hair and nails for two to four weeks prior to data collection. About 200-300 strands of occipital hair from hair root was cut by trained sampling personnel. In addition, the participant was asked to clip about 1 mm of all hand and foot nails. The hair and nail samples were placed in separate aluminum foils in codified Ziplock bags with added humidity absorber and then were stored in room temperature.

**S1 Box. Biospecimen ascertainment**
