# Supplementary figures and images for: Alarm of non-communicable disease in Iran: Kavar cohort profile, baseline and 18-month follow up results from a prospective population-based study in urban area
Source: PLoS One. 2022 Jan 27;17(1):e0260227. doi: 10.1371/journal.pone.0260227 (PMC8794109; doi:10.1371/journal.pone.0260227)

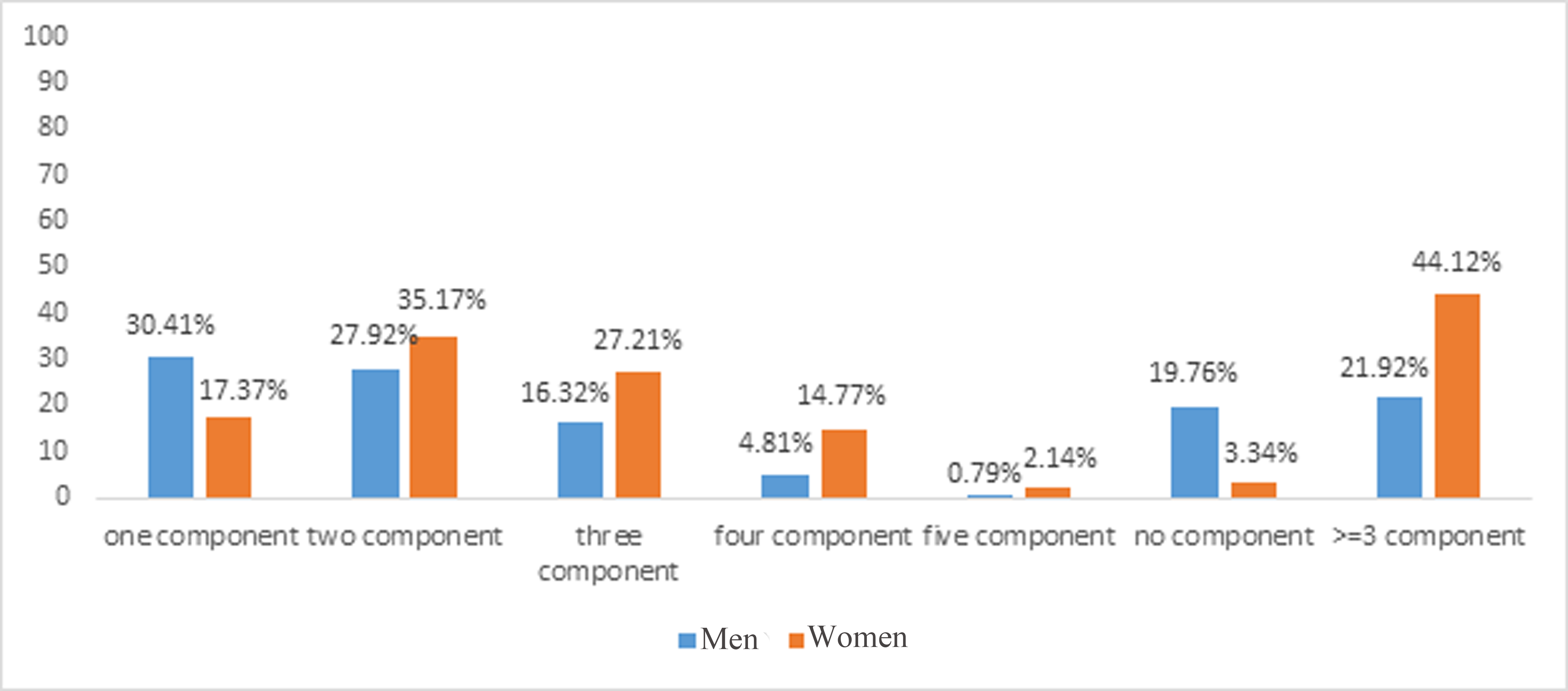

Supplement: S1 Fig — (TIF) [file pone.0260227.s002.tif]
