# Supplementary material for: Alarm of non-communicable disease in Iran: Kavar cohort profile, baseline and 18-month follow up results from a prospective population-based study in urban area
Source: PLoS One. 2022 Jan 27;17(1):e0260227. doi: 10.1371/journal.pone.0260227 (PMC8794109; doi:10.1371/journal.pone.0260227)
Supplement: S1 Table — (DOCX) [file pone.0260227.s003.docx]

**S1 Table. Biochemical parameters at the primary phase**

| **Lab test** | **Unit** | **Men**  **mean (SD)** | **Women**  **mean (SD)** | **P-Value** |
| --- | --- | --- | --- | --- |
| **HGB** | g/dl | 15.51 (1.42) | 13.57 (1.37) | <0.001 |
| **FBS** | Mg/dl | 100.95 (30.78) | 102.81 (33.40) | 0.041 |
| **BUN** | Mg/dl | 30.87 (7.87) | 26.96 (7.74) | <0.001 |
| **Creatinine** | Mg/dl | 1.19 (0.18) | 0.95 (0.15) | <0.001 |
| **TG** | Mg/dl | 159.74 (103.61) | 141.42 (79.55) | <0.001 |
| **CHOL** | Mg/dl | 171.49 (37.95) | 178.53 (36.12) | <0.001 |
| **SGOT** | Iu/l | 19.78 (11.31) | 16.73 (6.84) | <0.001 |
| **SGPT** | Iu/l | 24.09 (15.88) | 17.03 (9.84) | <0.001 |
| **ALP** | Iu/l | 202.23 (55.71) | 195.13 (60.55) | <0.001 |
| **HDL** | Mg/dl | 39.12 (8.37) | 44.90 (9.61) | <0.001 |
| **GGT** | Iu/l | 27.53 (20.91) | 21.40 (17.34) | <0.001 |

Abbreviations: HGB, Hemoglobin; FBS, Fasting blood sugar; BUN, Blood urea nitrogen; TG, Triglyceride; CHOL, Total cholesterol; SGOT, Serum Glutamic-Oxaloacetic Transaminase; SGPT, Serum glutamate pyruvate transaminase; ALP, Alkaline phosphatase; HDL, High density lipoprotein; GGT, Gamma glutamyl transpeptidase.
