# Supplementary material for: Alarm of non-communicable disease in Iran: Kavar cohort profile, baseline and 18-month follow up results from a prospective population-based study in urban area
Source: PLoS One. 2022 Jan 27;17(1):e0260227. doi: 10.1371/journal.pone.0260227 (PMC8794109; doi:10.1371/journal.pone.0260227)
Supplement: S2 Table — (DOCX) [file pone.0260227.s004.docx]

**S2 Table. The self-report prevalence of NCDs at the primary phase**

| **NCDs** | **Men n (%)** | **Women n (%)** | **Total n (%)** | ***P-Value** |
| --- | --- | --- | --- | --- |
| **Kidney stone** | | | | |
| Yes | 748 (14.97%) | 682 (13.65%) | 1430 (28.62%) | <0.001 |
| No | 1671 (33.45%) | 1895 (37.93%) | 3566 (71.38%) |  |
| **Hypertension** | | | | |
| Yes | 324 (6.49%) | 607 (12.15%) | 931 (18.63%) | <0.001 |
| No | 2095 (41.93%) | 1970 (39.43%) | 4065(81.37%) |  |
| **Diabetes** | | | | |
| Yes | 285 (5.70%) | 498 (9.97%) | 783 (15.67%) | <0.001 |
| No | 2134 (42.71%)) | 2079 (41.61% | 4213 (84.33%) |  |
| **Fatty liver** | | | | |
| Yes | 247 (4.94%) | 504 (10.09%) | 751 (15.03%) | <0.001 |
| No | 2172 (43.47%) | 2073 (41.49%) | 4245 (84.97%) |  |
| **Thyroid disorders** | | | | |
| Yes | 106 (2.12%) | 510 (10.21%) | 616 (12.33%) | <0.001 |
| No | 2313 (46.30%) | 2067 (41.37%) | 4380 (87.67%) |  |
| **Ischemic heart disease** | | | | |
| Yes | 195 (3.90%) | 149 (2.98%) | 344 (6.89%) | 0.001 |
| No | 2224 (44.52%) | 2428 (48.60%) | 4652 (93.11%) |  |
| **Rheumatic diseases** | | | | |
| Yes | 65 (1.30%) | 188 (3.76%) | 253 (5.06%) | <0.001 |
| No | 2354 (47.12%) | 2389 (47.82%) | 4743 (94.94%) |  |
| **Gallstone** | | | | |
| Yes | 53 (1.06%) | 181 (3.62%) | 234 (4.68%) | <0.001 |
| No | 2366 (47.36%) | 2396 (47.96%) | 4762 (95.32%) |  |
| **Chronic lung disease (asthma, tuberculosis)** | | | | |
| Yes | 63 (1.26%) | 97 (1.94%) | 160 (3.20%) | 0.020 |
| No | 2356 (47.16%) | 2480 (49.64%) | 4836 (96.80%) |  |
| **Myocardial infarction** | | | | |
| Yes | 40 (0.8%) | 17 (0.34%) | 57 (1.14%) | <0.001 |
| No | 2379 (47.62%) | 2560 (51.24%) | 4939 (98.86%) |  |
| **Stroke** | | | | |
| Yes | 26 (0.52%) | 23 (0.46%) | 49 (0.98) | 0.513 |
| No | 2393 (47.90%) | 2554 (51.12%) | 4947 (99.02%) |  |
| **Cancer (Total)** | | | | |
| Yes | 9 (0.18%) | 30 (0.6%) | 39 (0.78%) | 0.001 |
| No | 2410 (48.24%) | 2547 (50.98%) | 4957 (99.22%) |  |
| **Breast Cancer** | | | | |
| Yes | 0 | 16 (0.32%) | 16 (0.32%) | <0.001 |
| No | 2419 (48.42%) | 2561 (51.26%) | 4980 (99.68%) |  |
| **CNS Cancer** | | | | |
| Yes | 0 | 6 (0.12%) | 6 (0.12%) | 0.032 |
| No | 2419 (48.42%) | 2571 (51.46%) | 4990 (99.88%) |  |
| **Skin Cancer** | | | | |
| Yes | 1 (0.02%) | 0 | 1 (0.02%) | 0.484 |
| No | 2418 (48.40%) | 2577 (51.58%) | 4995 (99.98%) |  |
| **Hematopoietic System Cancer** | | | | |
| Yes | 1 (0.02%) | 2 (0.04%) | 3 (0.06%) | 0.601 |
| No | 2418 (48.40%) | 2575 (51.54%) | 4993 (99.94%) |  |
| **Uterine Cancer** | | | | |
| Yes | 0 | 3 (0.06%) | 3 (0.06%) | 0.251 |
| No | 2419 (48.42%) | 2574 (51.52%) | 4993 (99.94%) |  |
| **Colorectal Cancer** | | | | |
| Yes | 2 (0.04%) | 1 (0.02%) | 3 (0.06%) | 0.527 |
| No | 2417 (48.38%) | 2576 (51.56%) | 4993 (99.94%) |  |
| **Bladder Cancer** | | | | |
| Yes | 2 (0.04%) | 0 | 2 (0.04%) | 0.234 |
| No | 2417 (48.38%) | 2577 (51.58%) | 4994 (99.96%) |  |
| **Esophagus Cancer** | | | | |
| Yes | 1 (0.02%) | 0 | 1 (0.02%) | 0.484 |
| No | 2418 (48.40%) | 2577 (51.58%) | 4995 (99.98%) |  |
| **Prostate Cancer** | | | | |
| Yes | 1 (0.02%) | 0 | 1 (0.02%) | 0.484 |
| No | 2418 (48.40%)) | 2577 (51.58% | 4995 (99.98%) |  |
| **Lung Cancer** | | | | |
| Yes | 0 | 1 (0.02%) | 1 (0.02%) | 1.000 |
| No | 2419 (48.42%) | 2576 (51.56%) | 4995 (99.98%) |  |
| **Laryngeal Cancer** | | | | |
| Yes | 1 (0.02%) | 0 | 1 (0.02%) | 0.484 |
| No | 2418 (48.40%) | 2577 (51.58%) | 4995 (99.98%) |  |
| **Laryngeal Cancer** | | | | |
| Yes | 0 | 1 (0.02%) | 1 (0.02%) | 1.000 |
| No | 2419 (48.42%) | 2576 (51.56%) | 4995 (99.98%) |  |

* The results of chi squared and Fisher Exact tests.
