# Supplementary material for: Alarm of non-communicable disease in Iran: Kavar cohort profile, baseline and 18-month follow up results from a prospective population-based study in urban area
Source: PLoS One. 2022 Jan 27;17(1):e0260227. doi: 10.1371/journal.pone.0260227 (PMC8794109; doi:10.1371/journal.pone.0260227)
Supplement: S3 Table — (DOCX) [file pone.0260227.s005.docx]

**S3 Table. Association of demographic and lifestyle variables with non-communicable disease at the start of study in 2419 Men**

| **Parameters** | **Diabetes**  **N=285(11.8%)** | ***P- Value** | | **Hypertension**  **N=324(13.4%)** | ***P-Value** | **IHD**  **N=193(7.9%)** | ***P- Value** |
| --- | --- | --- | --- | --- | --- | --- | --- |
| **Age group** | | | | | | | |
| 35-50 | 99(34.7%) | <0.001 | | 93(28.7%) | <0.001 | 48(24.60%) | <0.001 |
| 51-60 | 120(42.13%) |  |  | 131(40.4%) |  | 84(43.10%) |  |
| 61-70 | 66(23.23%) |  |  | 100(30.9%) |  | 63(32.30%) |  |
| **Education** | | | | | | | |
| Illiterate | 74(26.12%) | <0.001 | | 85(26.2%) | 0.004 | 57(29.20%) | 0.007 |
| Elementary | 139(48.82%) |  |  | 146(45.1%) |  | 89(45.60%) |  |
| High school | 43(15.33%) |  |  | 45(13.9%) |  | 28(14.40%) |  |
| University | 29(10.24%) |  |  | 48(14.8%) |  | 21(10.80%) |  |
| **Marital status** | | | | | | | |
| Single | 0 | 0.06 | | 2(0.6%) | <0.001 | 0 | 0.03 |
| Married | 284(99.62%) |  |  | 321(99.1%) |  | 194(99.50%) |  |
| Widowed | 0 |  |  | 1(0.3%) |  | 0 |  |
| Divorced | 1(0.38%) |  |  | 0(0%) |  | 1(0.50%) |  |
| **BMI** | | | | | | |  |
| Underweight | 3 (1.12%) | <0.001 | | 5 (1.50%) | <0.001 | 3(1.50%) | <0.001 |
| Normal | 96(33.75%) |  |  | 77 (23.8%) |  | 54(27.70%) |  |
| Overweight | 120 (42.10%) |  |  | 148 (45.70%) |  | 94(48.20%) |  |
| Obese | 66(23.23%) |  |  | 94 (29.0%) |  | 44(22.60%) |  |
| **Alcohol drinking(Current)** | | | | | | | |
| Yes | 25 (8.77%) | | <0.001 | 37(11.41%) | 0.001 | 25(12.80%) | 0.06 |
| No | 260 (91.23%) | |  | 287 (88.59%) |  | 170(87.20%) |  |
| **Smoking status** | | | | | | | |
| Yes | 74 (26.00%) | | 0.26 | 141 (43.50%) | 0.25 | 97(49.70%) | 0.36 |
| No | 211 (74.00%) | |  | 183 (56.50%) |  | 98(50.30%) |  |
| **hypercholesterolemia** | | | | | | | |
| Yes(>=240mg/dl) | 16(5.70%) | | 0.27 | 13(4.00%) | 0.88 | 5(2.60%) | <0.001 |
| No(<240mg/dl) | 265(94.30%) | |  | 309(96.00%) |  | 188(97.40%) |  |

*Results are from chi squared test or Fisher exacted test.
