# Supplementary material for: Alarm of non-communicable disease in Iran: Kavar cohort profile, baseline and 18-month follow up results from a prospective population-based study in urban area
Source: PLoS One. 2022 Jan 27;17(1):e0260227. doi: 10.1371/journal.pone.0260227 (PMC8794109; doi:10.1371/journal.pone.0260227)
Supplement: S4 Table — (DOCX) [file pone.0260227.s006.docx]

**S4 Table. Association of demographic and lifestyle variables with non-communicable disease at the start of study in 2578 women**

| **Parameters** | **Diabetes**  **N= 497 (%)** | ***P- Value** | | **Hypertension**  **N= 607(%)** | ***P-Value** | **IHD**  **N= 149(%)** | ***P- Value** |
| --- | --- | --- | --- | --- | --- | --- | --- |
| **Age group** | | | | | | | |
| 35-50 | 194(39.00%) | <0.001 | | 227(37.40%) | 0.004 | 41(27.50%) | 0.007 |
| 51-60 | 189(38.00%) |  |  | 231(38.10%) |  | 54(36.20%) |  |
| 61-70 | 114(23.00%) |  |  | 149(24.50%) |  | 54(36.20%) |  |
| **Education** | | | | | | | |
| Illiterate | 310(62.40%) | <0.001 | | 374(61.60%) | <0.001 | 100(67.10%) | <0.001 |
| Elementary | 163(32.80%) |  |  | 196(32.30%) |  | 46(30.90%) |  |
| High school | 16(3.20%) |  |  | 30(4.90%) |  | 3(2.00%) |  |
| University | 8(1.60%) |  |  | 7(1.20%) |  | 0 |  |
| **Marital status** | | | | | | | |
| Single | 12(24%) | <0.001 | | 7(1.20%) | <0.001 | 0 | <0.001 |
| Married | 406(81.7%) |  |  | 495(81.50%) |  | 119(79.90%) |  |
| Widowed | 70(14.10%) |  |  | 101(16.60%) |  | 30(20.10%) |  |
| Divorced | 9(1.80) |  |  | 4(0.70%) |  | 0 |  |
| **BMI** | | | | | | |  |
| Underweight | 1 (0.2%) | <0.001 | | 2 (0.30%) | <0.001 | 2(1.30%) | 0.05 |
| Normal | 78(15.70%) |  |  | 76 (12.50%) |  | 16(10.70%) |  |
| Overweight | 185(37.2%) |  |  | 221 (36.40%) |  | 60(40.30%) |  |
| Obese | 233(46.90%) |  |  | 308 (50.70%) |  | 71(47.70%) |  |
| **Alcohol drinking(Current)** | | | | | | | |
| Yes | 0(0%) | | <0.001 | 1(0.20%) | 0.66 | 0 | 0.74 |
| No | 497 (100.00%) | |  | 606(99.80%) |  | 149(100%) |  |
| **Smoking status** | | | | | | | |
| Yes | 123 (24.70%) | | 0.04 | 13 (2.13%) | 0.04 | 3837 (76.82%) | 0.12 |
| No | 374(75.30%) | |  | 594 (97.87%) |  | 775 (15.52%) |  |
| **hypercholesterolemia** | | | | | | | |
| Yes(>=240mg/dl) | 40(7.80%) | | 0.03 | 41(7.00%) | 0.15 | 12(8.30%) | 0.20 |
| No(<240mg/dl) | 457(92.20%) | |  | 548(93.00%) |  | 132(91.70%) |  |

*Results are from chi squared test or Fisher exacted test.
