# Supplementary material for: Alarm of non-communicable disease in Iran: Kavar cohort profile, baseline and 18-month follow up results from a prospective population-based study in urban area
Source: PLoS One. 2022 Jan 27;17(1):e0260227. doi: 10.1371/journal.pone.0260227 (PMC8794109; doi:10.1371/journal.pone.0260227)
Supplement: S5 Table — (DOCX) [file pone.0260227.s007.docx]

**S5 Table. New cases of non-communicable diseases after 18 months of follow-up at the time of registration**

| **Outcomes after 18 months^*^** | **Men**  **n (%)** | **Women**  **n (%)** | **Total**  **n (%)** |
| --- | --- | --- | --- |
| **Hypertension** | 63  (1.26%) | 53  (1.06%) | 116  (2.32%) |
| **Diabetes** | 18  (0.36%) | 14  (0.28%) | 32  (0.64%) |
| **Cardiovascular outcomes** | 27  (0.54%) | 6  (0.12%) | 33  (0.66%) |
| **Cerebrovascular outcomes** | 1  (0.02%) | 4  (0.08%) | 5  (0.1%) |
| **Asthma** | 0 | 3  (0.06%) | 3  (0.06%) |
| **Chronic kidney disease**  **(dialysis)** | 2  (0.04%) | 0 | 2  (0.04%) |
| **Lymph node cancer** | 2  (0.04%) | 0 | 2  (0.04%) |
| **Colorectal cancer** | 0 | 1  (0.02%) | 1  (0.02%) |
| **Parkinson’s disease** | 1  (0.02%) | 0 | 1  (0.02%) |
| **Breast**  **cancer** | 0 | 1  (0.02%) | 1  (0.02%) |
| **Pancreatic**  **cancer** | 0 | 1  (0.02%) | 1  (0.02%) |
| **Brain**  **tumor** | 0 | 1  (0.02%) | 1  (0.02%) |

*These outcomes were recorded and confirmed according to ICD-10 codes
