# Supplementary material for: Alarm of non-communicable disease in Iran: Kavar cohort profile, baseline and 18-month follow up results from a prospective population-based study in urban area
Source: PLoS One. 2022 Jan 27;17(1):e0260227. doi: 10.1371/journal.pone.0260227 (PMC8794109; doi:10.1371/journal.pone.0260227)
Supplement: S6 Table — (DOCX) [file pone.0260227.s008.docx]

**S6 Table. Association of demographic and life style variables with outcomes**

| **Parameters** | **Diabetes out come**  **N=31(0.6%)** | ***P- Value** | | **Hypertension out come**  **N=116(2.3%)** | ***P-Value** | **IHD** out come**  **N=25(0.5%)** | ***P- Value** |
| --- | --- | --- | --- | --- | --- | --- | --- |
| **Gender** | | | | | | | |
| Men | 17(54.80%) | 0.47 | | 63(54.30%) | 0.19 | 20(80.00%) | 0.002 |
| Women | 14(45.20%) |  |  | 53(45.7%) |  | 5(20.00%) |  |
| **Age group** | | | | | | | |
| 35-50 | 16(51.60%) | 0.26 | | 52(44.80%) | <0.001 | 7(28.00%) | 0.002 |
| 51-60 | 12(38.70%) |  |  | 43(37.10%) |  | 13(52.00%) |  |
| 61-70 | 3(9.70%) |  |  | 21(18.10%) |  | 5(20.00%) |  |
| **Education** | | | | | | | |
| Illiterate | 11(35.50%) | 0.40 | | 50(43.10%) | 0.01 | 7(28.00%) | 0.04 |
| Elementary | 15(48.40%) |  |  | 48(41.40%) |  | 7(28.00%) |  |
| High school | 1(3.20%) |  |  | 7(6%) |  | 6(24.00%) |  |
| University | 4(12.90%) |  |  | 11(9.50%) |  | 5(20.00%) |  |
| **Marital status** | | | | | | | |
| Single | 0 | 0.72 | | 0 | 0.04 | 0 | 0.80 |
| Married | 29(93.50%) |  |  | 106(91.40%) |  | 24(96.00%) |  |
| Widowed | 0 |  |  | 6(5.20%) |  | 1(4.00%) |  |
| Divorced | 2(6.5%) |  |  | 4(3.40%) |  | 0 |  |
| **BMI** | | | | | | |  |
| Underweight | 0 | 0.15 | | 2(1.70%) | 0.07 | 0 | 0.52 |
| Normal | 4(12.91%) |  |  | 22(19.00%) |  | 5(20.00%) |  |
| Overweight | 15(48.40%) |  |  | 53(45.70%) |  | 13(52.00%) |  |
| Obese | 12(38.7%) |  |  | 39(33.60%) |  | 7(28.00%) |  |
| **Alcohol drinking(Current)** | | | | | | | |
| Yes | 0 | | 0.08 | 4(3.40%) | 0.04 | 2(8.00%) | 0.86 |
| No | 31(100%) | |  | 112(96.60%) |  | 23(92.00%) |  |
| **Smoking status** | | | | | | | |
| Yes | 8 (25.80%) | | 0.73 | 22(19.00%) | 0.27 | 10(40.00%) | 0.04 |
| No | 23 (74.20%) | |  | 94(81.00%) |  | 15(60.00%) |  |
| **hypercholesterolemia** | | | | | | | |
| Yes(>=240mg/dl) | 6(13.80%) | | 0.03 | 8(6.10%) | 0.62 | 6(24.00%) | <0.001 |
| No(<240mg/dl) | 25(86.20%) | |  | 108(93.90%) |  | 19(76.00%) |  |

*Results from Chi squared or Fisher exacted tests.

**Ischemic Heart Disease
