# Supplementary material for: Alarm of non-communicable disease in Iran: Kavar cohort profile, baseline and 18-month follow up results from a prospective population-based study in urban area
Source: PLoS One. 2022 Jan 27;17(1):e0260227. doi: 10.1371/journal.pone.0260227 (PMC8794109; doi:10.1371/journal.pone.0260227)
Supplement: S7 Table — (DOCX) [file pone.0260227.s009.docx]

**S7 Table. Mortality by cause during 18 months of follow-up in PKCS**

| **Cause of**  **Death^*^** | **Men**  **n (%)** | **Women**  **n (%)** | **Total**  **n (%)** |
| --- | --- | --- | --- |
| **Sudden cardiac death** | 2(0.04%) | 4(0.08%) | 6(0.12%) |
| **Intra-cerebral hemorrhage** | 0 | 1(0.02%) | 1(0.02%) |
| **Brain injury** | 1(0.02%) | 0 | 1(0.02%) |
| **Myocardial infarction** | 1(0.02%) | 2(0.04%) | 3(0.06%) |
| **Breast cancer** | 0 | 2(0.04%) | 2(0.04%) |
| **Chronic kidney disease** | 0 | 1(0.02%) | 1(0.02%) |
| **Suicide** | 1(0.02%) | 0 | 1(0.02%) |
| **Fall from cliff** | 1(0.02%) | 0 | 1(0.02%) |
| **Total** | 6(0.12%) | 10(0.20 %) | 16(0.32%) |

^*^These causes were recorded and confirmed according to ICD-10 codes
